# Oxyphylla A Promotes Degradation of α-Synuclein for Neuroprotection *via* Activation of Immunoproteasome

**DOI:** 10.14336/AD.2019.0612

**Published:** 2019-06-12

**Authors:** Hefeng Zhou, Shengnan Li, Chuwen Li, Xuanjun Yang, Haitao Li, Hanbing Zhong, Jia-Hong Lu, Simon Ming-Yuen Lee

**Affiliations:** ^1^State Key Laboratory of Quality Research in Chinese Medicine and Institute of Chinese Medical Sciences, University of Macau, Macao, China.; ^2^Department of Biology, South University of Science and Technology, Shenzhen, China.

**Keywords:** Parkinson’s disease, α-synuclein, degradation, proteasome, neuroprotection

## Abstract

Parkinson’s disease (PD), the second most common neurodegenerative disorder, is neuropathologically characterized by the loss of dopaminergic neurons in the *substantia nigra pars compacta* (SNc) and the presence of Lewy bodies in surviving neurons. α-synuclein (α-syn) is the major component of Lewy bodies and its deposition in neurons is critical pathological event in the pathogenesis of PD. Herein, we reported that Oxyphylla A, a novel lead compound from the fruit of *Alpinia oxyphylla*, significantly promoted α-syn degradation in a cellular PD model. When exploring the molecular pathways, we found that Oxyphylla A promoted α-syn degradation in a ubiquitin proteasome system (UPS)-dependent and autophagy-independent manner. We further confirmed that Oxyphylla A enhanced UPS activity by upregulating 20S subunit PSMB8 expression. A mechanism study revealed that Oxyphylla A activated the PKA/Akt/mTOR pathway to trigger PSMB8 expression and enhance UPS activity. Finally, we illustrated that Oxyphylla A alleviated the accumulation of both Triton-soluble and Triton-insoluble forms of α-syn and protected against α-syn-induced neurotoxicity in A53T α-syn transgenic mice. These findings suggest that the activation of UPS, via small molecular UPS enhancers including Oxyphylla A, may be a therapeutic strategy for intervention against PD and related diseases.

As the second most common neurodegenerative disorder, PD is pathologically characterized by the loss of dopaminergic cells in the *substantia nigra pars compacta* (SNc) and the presence of Lewy bodies [1, 2]. Alpha-synuclein (α-syn) is the main component of Lewy bodies and abnormalities in α-syn represent one of the major factors in PD development [3]. Previous studies show that natively unfolded α-syn becomes naturally folded through the process of its oligomerization and aggregation [4]. Genetic studies have found that some point mutations in the SNCA gene lead to the early-onset (A30P, E46K, G51D, A53E and A53T) or late-onset (H50Q) forms of familial PD [5-7]. Among these mutations, A53T α-syn can significantly enhance protein oligomerization, leading to α-syn aggregation, Lewy body formation and neurotoxicity [8]. Furthermore, duplications and triplications of the SNCA gene have also been associated with PD [9, 10]. The overexpression of α-syn in Tg mice models can result in progressive locomotor defects with dopaminergic neuron loss and α-syn aggregation in the brain [11-13]. Failure to clear high amounts of α-syn accelerates PD progression [14]. Therefore, developing therapeutic approaches for targeting α-syn could be useful and practical for PD treatment [15, 16].

Currently, much attention has been focused on eliminating α-syn toxicity via degradation through the ubiquitin-proteasome system (UPS) and the autophagy-lysosome pathway (ALP) [17, 18]. Many findings have indicated that clearance of α-syn accumulation by these two proteolytic pathways plays a pivotal role in numerous neurodegenerative conditions. The UPS is responsible for the highly selective degradation of mostly short-lived and soluble proteins under basal metabolic conditions, as well as misfolded or damaged proteins in the cytosol, nucleus or endoplasmic reticulum [19, 20]. In contrast, ALP achieves vesicle-mediated degradation of longer-lived macromolecules, dysfunctional organelles and cytosolic components through three main autophagic pathways: chaperone-mediated autophagy (CMA), macroautophagy, and microautophagy [21]. To our knowledge, strong interplay has been observed between the degradation of α-syn and the UPS or autophagic pathways, except in the case of microautophagy. Previous reports have proven that UPS could degrade certain specific species of α-syn, such as small, soluble oligomers [22]. CMA has shown a specific capacity to degrade monomers and dimers of α-syn [22]. Some studies have shown that soluble oligomers of α-syn could cause 26S proteasome dysfunction [23, 24], while two α-syn familial mutations, A30P and A53T, could cause CMA degradation, leading to an increase in toxicity in cells [25, 26]. Additionally, WT and mutated α-syn can be degraded through macroautophagy, but blockade of this pathway generally leads to the accumulation of a high molecular weight form of α-syn [27]. Intriguingly, recent α-syn Tg mouse models have shown that UPS can degrade α-syn under conditions of endogenous and increased protein burden, whereas ALP took over in pathologic conditions when α-syn levels were elevated [28]. Moreover, functional cross-talk between the UPS and the ALP has been shown to modulate intracellular α-syn levels in the context of dysregulation [29]. Therefore, the dysfunction of these degradation pathways may be a critical factor contributing to α-syn accumulation and, thus, to PD pathogenesis. The modulation of α-syn protein levels *via* these degradation pathways has a potential as a therapeutic intervention.

As is well known, current symptomatic treatments fail to treat all symptoms of PD and do not alter the synuclein neuropathology [30]. Traditional Chinese medicines (TCMs) have anti-aging and neuroprotective abilities, rendering them alternative strategies for treating neurodegenerative disorders [31, 32]. Many natural compounds in herbal medicines display a promising neuroprotective effect in cells and animals, such as curcumin, trehalose and kaempferol [33].

Oxyphylla A ((R)-4-(2-hydroxy-5-methylphenyl)-5-methylhexanoic acid), a novel bioactive compound of Chinese herb *Alpinia oxyphylla*, was previously identified as a promising anti-PD compound by our group [34, 35]. One has been reported that Oxyphylla A ameliorates chemical-induced primary neuronal cell damage and behavioral impairment both zebrafish and C57BL/6 mouse PD models [34]. The other previous work showed that the ethanolic extract of Fructus Alpinia oxyphylla (AOE) prevented and restored 6-hydroxydopamine (6-OHDA)-induced dopaminergic (DA) neuron degeneration involving PI3K-AKT pathway [35]. These results suggested that Oxyphylla A displayed a promising neuroprotective e?ects both *in vitro* and *in vivo*. Herein, further functional and pathological studies were carried out.

In this present study, we revealed the protective effect of Oxyphylla A against A53T α-syn-induced neurotoxicity, both *in vitro* and *in vivo*. Oxyphylla A was able to selectively promote the degradation of WT and mutant α-syn *via* the UPS by upregulating the expression of *PSMB8*. Furthermore, the PKA/Akt/mTOR pathway was also identified as a mechanism underlying the Oxyphylla A-driven A53T α-syn degradation. We envisage that Oxyphylla A could be used as a starting structural template for further development of more potent small molecules, to promote α-syn degradation for treatment of PD.

## MATERIALS AND METHODS

### Reagents and antibodies

Doxycycline (Dox) (D9891), chloroquine (CQ) (C6628), dimethyl sulfoxide (DMSO) (D2650), paraformaldehyde (PFA) (16005), phenylmethanesulfonyl fluoride (PMSF) (P7626) and thiazolyl bluet tetrazolium bromide (MTT) (M2128) were purchased from Sigma-Aldrich (St. Louis, MO, USA). A lactate dehydrogenase (LDH) kit (11644793001) and protease inhibitor cocktail (11836170001) were purchased from Roche Applied Science (Indianapolis, IN, USA). F-12K medium (21127022), fetal bovine serum (FBS) (26140079), horse serum (HS) (26050088), penicillin-streptomycin (PS) (10378016) , trypsin-EDTA (11560626), and phosphate-buffered saline (PBS) (10316743) were purchased from Life Technologies (Grand Island, NY, USA). Enhanced chemiluminescence (ECL) solution (10308449) was obtained from Thermo Scientific (Rockford, IL, USA). H-89 (S1582) and MG132 (S2619) were purchased from Selleck Chemicals (Shanghai, China). SYBR^®^ Premix Ex Taq™ II kit (RR820A) was purchased from TaKaRa (Dalian, China). Antibodies against α-syn (#2642), p-PKA (#5661), PKA (#5842), p-Akt (#4060), Akt (#2920), p-mTOR (#5536), mTOR (#2983), p-p70S6K (#9205), p70S6K (#2708), LC3A/B (#12741), SQSTM1/p62 (#39749), PSMB8 (#13726), β-actin (#8457), HRP-conjugated anti-mouse IgG (#3700) and anti-rabbit IgG (#7074) were purchased from Cell Signaling Technology (Boston, MA, USA). Proteasome-Glo™ 3-Substrate Cell-Based Assay System (G1180) was purchased from Promega (Madison, WI, USA). TRIzol reagent (15596026) were purchased from life technologies (Eugene, OR, USA). Oxyphylla A was synthesized in our laboratory. All other chemicals of analytical grade were purchased from local sources.

### Cell culture and treatments

Inducible PC12 stable cell lines overexpressing α-syn (WT, A30P, A53T), as previously characterized [22], were maintained in 150 μg/ml hygromycin B in DMEM supplemented with 10% HS, 5% FBS, 100 units/ml penicillin/streptomycin, and 50 μg/ml G418 in a humidified atmosphere of 5% CO_2_ and 95% air at 37°C. Inducible PC12 cells were treated with 1 μg/ml Dox to induce the expression of α-syn for 24 h and then washed twice with medium to remove Dox. Then cells were incubated in media containing either Oxyphylla A (100 μM), CQ (30 μM), MG132 (0.1 μΜ) or DMSO for 24 h. Oxyphylla A was dissolved in DMSO at 100 mM as a stock solution and added directly to the culture media to a final working concentration of 0.1% (v/v) DMSO.

### Cell viability and cytotoxicity analysis

Inducible PC12/A53T-α-syn cells were seeded in 96-well plates at a density of 8?×?10^3^ cells/well for 24 h. After treatment with different concentrations of Oxyphylla A (ranging from 10-300 µM) for 24 h, the supernatant was removed for LDH cytotoxicity analysis, while the remainder was used for MTT cell viability analysis. Absorbance was measured at 490 nm for LDH and 570 nm for MTT using a SpectraMax M5 (Wallace, Netherlands).

### Proteasomal activity assay

The enzymatic activities of the chymotrypsin-, trypsin- and caspase-like proteasome were measured using the Proteasome-Glo™ 3-Substrate Cell-Based Assay System (Promega, USA) following the manufacturer’s instructions. Briefly, inducible PC12/A53T-α-syn cells were treated with or without 1 μg/ml Dox for 24 h, followed by treatment with 100 μΜ Oxyphylla A, 0.1 μΜ MG132 for another 24 h. The cells were harvested and lysed with Proteasome-Glo™ Cell-Based Reagent. Cell lysates were assayed in triplicate for each proteasome. Suc-LLVY aminoluciferin (succinyl-leucine-leucine-valine-tyrosine-aminoluciferin) substrate was used for the measurement of chymotryptic-like activity, while Z-LRR-aminoluciferin (Z-leucine-argininearginine-aminoluciferin) substrate was applied for trypsin-like activity detection. Z-nLPnLD-aminoluciferin (Z-norleucine-proline-norleucine-aspartate-aminoluciferin) substrate was then employed for the analysis of caspase-like activity. After reagents were added and mixed by plate shaking, luminescence was recorded using the SpectraMax M5.

### Extraction of Triton-soluble and Triton-insoluble fractions of α-syn protein

The Triton-soluble and Triton-insoluble fractions of α-syn protein from cell samples were sequentially extracted according to the published protocol with a slight modification [36]. Briefly, cells were washed twice with ice-cold PBS and then lysed in Triton lysis buffer (1% Triton X-100, 0.1 mM PMSF, 1% (v/v) protease inhibitor cocktail in PBS), vortexed for 10 sec, and incubated on ice for 20 min. Similarly, tissue samples from mouse brain were weighed and 100 mg samples were homogenized in five volumes of the same lysis buffer using tissue homogenizer. After homogenization, samples were rotated at 4?°C for 30?min for complete lysis. After centrifugation at 500 g for 10 min at 4 °C, the supernatants from tissue samples were collected. Then the cell samples and tissue samples were centrifuged at 18,000 g for 30 min at 4 °C. The supernatants were taken as Triton-soluble fractions. The pellets in cell samples and tissue samples were rinsed with Triton lysis buffer and re-centrifuged in order to remove any trace of the soluble fraction. The pellet was resuspended in 2% SDS-containing lysis buffer by pipetting and subsequent sonication for 10 seconds. The resultant solution represented the Triton-insoluble fractions.

### Western blotting analysis

Protein levels were examined using western blotting analysis as previously described [37]. Briefly, after appropriate treatment, the collected cells were lysed with RIPA lysis buffer [50?mM Tris-HCl pH 7.4, 150?mM NaCl, 1% NP-40, 5?mM EDTA, 0.5% sodium deoxycholate, 0.1% SDS, 1mM PMSF, 1× protease inhibitor cocktail]. For the brain samples, tissues were homogenized in RIPA lysis buffer to extract protein. Protein concentration was measured by a BCA protein assay kit. The same amounts of protein samples (20 µg) were electrophoresed on SDS-polyacrylamide gel and transferred to PVDF membrane. Membranes were subsequently incubated overnight at 4°C with various primary antibodies in 5% fat-free dry milk-TBST. Each antibody was diluted 1:1000: α-syn, phospho-PKA (Thr197), PKA, phospho-Akt (Ser473), Akt, phospho-mTOR (Ser2448), mTOR, phospho-p70S6K (Thr389), p70S6K, LC3A/B, and PSMB8, except β-actin (1:5000). The blots were then incubated with HRP-conjugated secondary antibody in TBST at a 1:5000 dilution for 1 h at room temperature. Protein bands were visualized with an ECL kit. The blot images were acquired with CCD-based imager. After development, the density of the bands was quantified by Image Lab Software (Bio-Rad, Hercules, USA).

### Quantitative real-time reverse transcription PCR (qRT-PCR) 

Total RNA was extracted from Oxyphylla A-treated PC12/A53T-α-syn cells by means of the TRIzol reagent. Each cDNA was synthesized using SuperScript II Reverse Transcriptase (Invitrogen) with random primers according to the manufacturer’s protocol. qRT-PCR was performed using the SYBR® Premix Ex Taq™ II kit and Mx3005P qPCR system (Agilent Technologies, Santa Clara, CA, USA). PCR reaction conditions were as follows: 5 min at 95 °C followed by 40 cycles of 95°C for 5 s, and 60 °C for 30 s. The relative mRNA level of each gene was normalized to GAPDH, which was used as an endogenous control. Primers used for the qRT-PCR analysis are listed in the supplementary Table 1.

### Construction of lentiviral vectors and lentivirus production

The full-length rat *PSMB8* cDNA was obtained by RT-PCR using total RNA extracted from PC12 cells and cloned into the pCDH-CMV-MCS-EF1-RFP lentiviral vector at NotI and XbaI sites. The primers used herein were 5′-GCTCTAGAGCCACCATGGCGTTACTGGA TCTG-3′ (sense) and 5′-TATGCGGCCGCTCACAGA GTGGCCTCTCGGT-3′ (antisense). A short-hairpin RNA (shRNA) designed against the rat *PSMB8* was cloned into the pSIH1-H1-DsRed vector at BamH I and EcoRI sites. The sequence of shRNA was GATCC GACCAGGAAAGGAAGGTTCAGCTCGAGCTGAACCTTCCTTTCCTGGTCTTTTTG; the mission non-target shRNA vector was used as a control. The identity of each construct was confirmed by sequencing. Plasmids were purified with the QiaPrep Miniprep kit (QIAGEN, IZASA, Portugal) and transfected into HEK293T lentiviral packaging cells with a three-plasmid system according to the manufacturer's protocols; lentiviral particles were harvested from the transfected cells after 48?h and 72 h. The lentiviruses were transfected into inducible PC12/A53T-α-syn cells. Transfection efficiency (approximately 80%) was calculated as percentage of transfected cells from all cells by counting transfected cells holding a red fluorescence protein (RFP) signal.

### Transgenic α-syn mouse model and treatment

Ten-month-old homozygous female transgenic mice B6 C3-Tg (Prnp-SNCA*A53T) 83Vle/J expressing A53T human α-syn were purchased from the Model Animal Research Center of Nanjing University (Nanjing, China). This well characterized Tg model was selected because of its severe movement impairment [38]. Mice were housed in standard cages with free access to food and water at 25°C following a 12 h: 12 h light/dark cycle. A53T human α-syn Tg mice (10 mice per group) received 0.2 ml Oxyphylla A (30 mg/kg) or olive oil as vehicle (0.2 ml) daily by gavage for 4 weeks. All animal experiments were approved by the Research Ethics Committee of Institute of Chinese Medical Sciences, University of Macau.

### Rotarod testing

Mice were first trained to stay on the rod of the rotarod, which was maintained at a constant speed (5 rpm) for at least 5 min. After training for 3 days, mice were tested for a total of three trials with a constant rotation speed of 22 rpm [39, 40]; the trial was started and then sustained for 10 min. The trial stopped when the mouse fell down (activating a switch that automatically stopped the timer) or when 10 min had elapsed.

### Immunohistochemistry

After Rotarod testing, mice were transcardially perfused with PFA in PBS (pH 7.4). Brains were dissected, post-fixed in PFA overnight and then dehydrated in 30% sucrose in PBS at 4°C. Sections were incubated in a monoclonal anti-TH primary antibody (1: 400, Millipore; MAB318) for 48 h at 4°C, washed in PBS, and incubated with secondary anti-rabbit-HRP antibody. Immuno-staining was visualized after 3-3’diamino-benzidine (DAB) staining (Vector Laboratories, USA) using bright field microscopy (Leica, Wetzlar, Germany). The number of TH-positive neurons with intact cell body and visible unstained nucleus was counted with Image J software (National Institutes of Health) by two blinded investigators. Five sections through the SNc areas (-5.3 mm AP from bregma) were selected from each animal. Values are expressed quantitatively as a percentage of the control group.

### Statistical analysis

Data and statistical analyses in this study complied with the recommendations on experimental design and analysis in pharmacology [41]. Statistical analyses were performed with one-way ANOVA followed by Tukey’s multiple comparison test (several groups) and unpaired Student's t-test (two groups) using GraphPad Prism 6.0 software (GraphPad Software Inc., San Diego, CA, USA), where a *P*-value less than 0.05 denoted statistical significance. Data are presented as means?±?SD.

**Figure 1.**
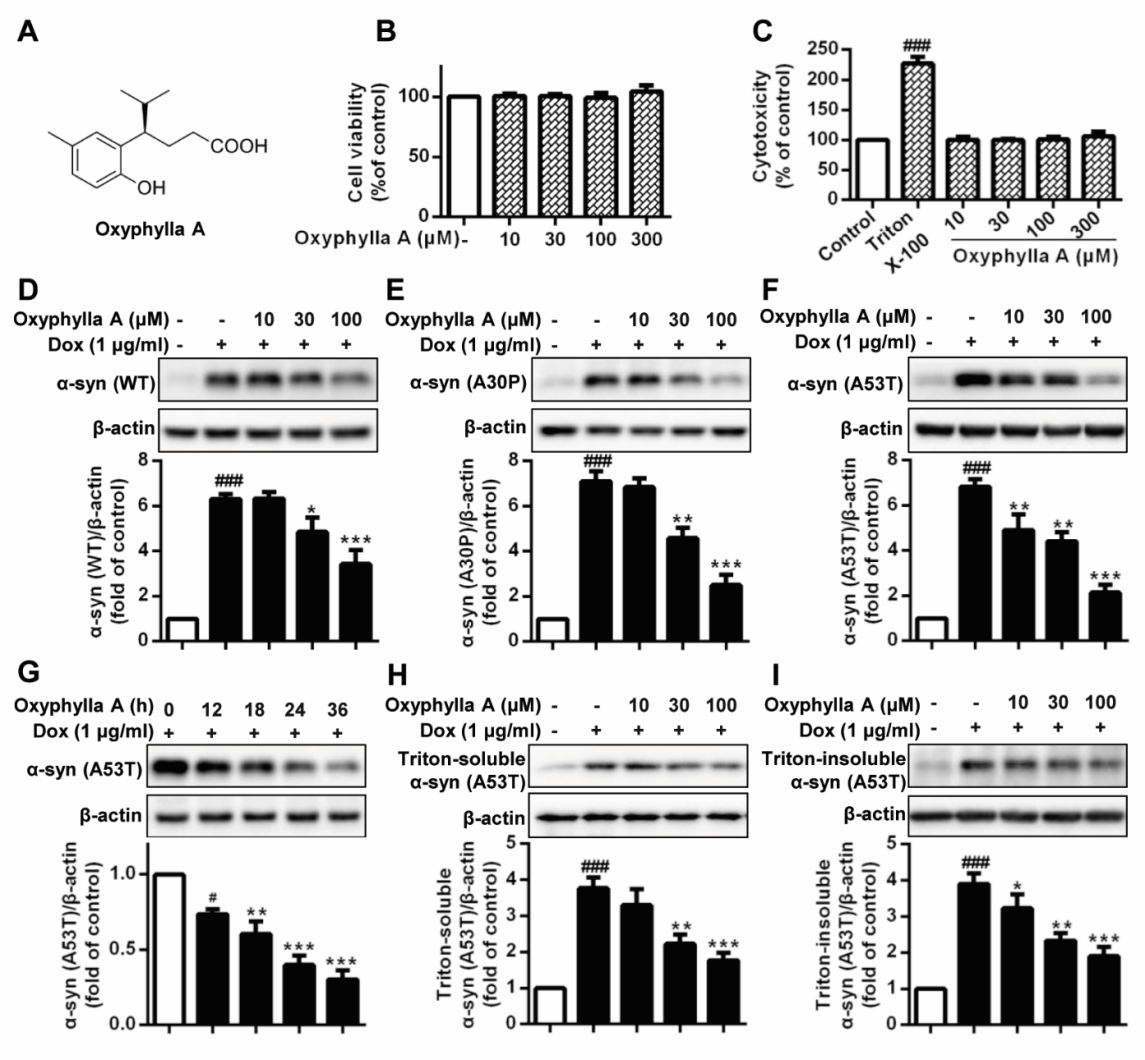
Oxyphylla A promotes the degradation of WT and mutant α-syn in cell models. (A) Chemical structure of Oxyphylla A. Inducible PC12/A53T-α-syn cells were treated with different concentrations of Oxyphylla A or 0.1% (v/v) DMSO for 24 h, and then analyzed by MTT assay (B) and LDH assay (C). Inducible PC12/WT-α-syn (D), A30P-α-syn (E) and A53T-α-syn (F) cells were treated with 1 μg/ml Dox for 24 h to induce the expression of WT or mutant α-syn, and then with different concentrations of Oxyphylla A or 0.1% (v/v) DMSO for another 24 h, cells were lysed in RIPA lysis buffer. (G) PC12/A53T-α-syn cells were induced with 1 μg/ml Dox for 24 h, and then treated with Oxyphylla A (100 μM) for different time periods. (H) After treatment with Oxyphylla A (100 μM), cells were lysed in 1% Triton X-100. The Triton-soluble fractions were subjected to Western blotting analysis. (I) Triton-insoluble fractions were also subjected to Western blotting analysis. Representative Western blotting and data analysis of three independent experiments are shown. Data are represented as the mean ± SD. ^###^*P* < 0.001 as compared to control group; *P < 0.05, **P < 0.01, ***P < 0.001, as compared to Dox group.

**Figure 2.**
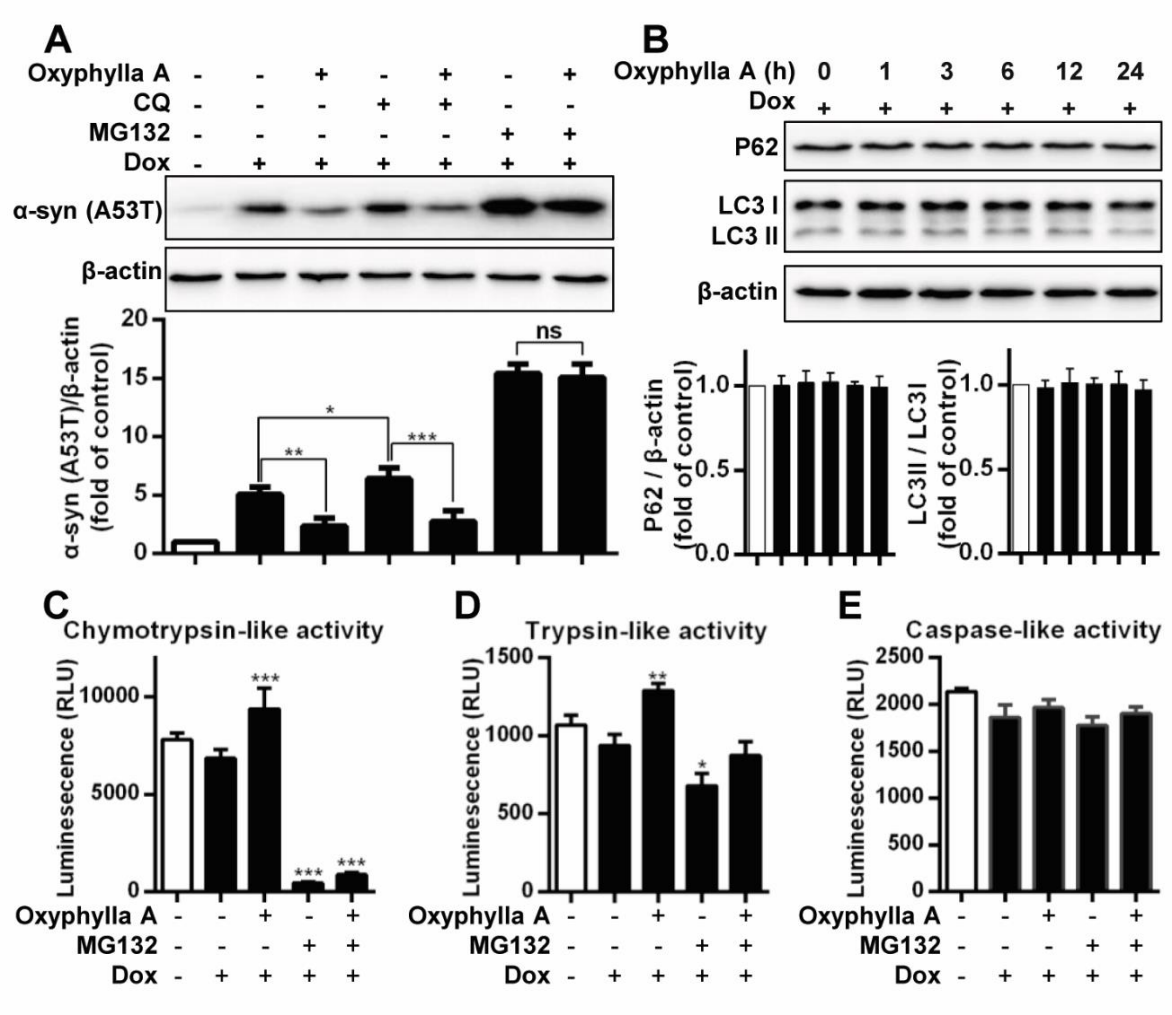
Oxyphylla A promotes α-syn degradation via the UPS. (A) Inducible PC12/A53T-α-syn cells were treated with or without 1 μg/ml Dox for 24 h, and then with 100 μΜ Oxyphylla A, 30 μΜ CQ, 0.1 μΜ MG132 or 0.1% (v/v) DMSO for another 24 h. (B) Inducible PC12/A53T-α-syn cells were treated with 1 μg/ml Dox for 24 h, and then treated with 100 μΜ Oxyphylla A for different time periods. Representative Western blotting and data analysis of three independent experiments are shown. The proteasomal enzyme activities were measured using specific bioluminogenic enzyme substrates for chymotrypsin-like (C), trypsin-like (D), and caspase-like (E) activity. Data from three independent experiments are represented as the mean ± SD. *P < 0.05, **P < 0.01, ***P < 0.001.

## RESULTS

### Oxyphylla A promotes the degradation of WT and mutant α-syn in PC12 cells

To examine the potential ability of Oxyphylla A (Fig. 1A) to degrade α-syn, Dox-inducible rat pheochromocytoma PC12 cell models overexpressing WT or mutant α-syn (A30P and A53T) were constructed. Preliminarily, we employed cell viability and cytotoxicity assays using inducible PC12/A53T-α-syn cells to measure compound neurotoxicity. After exposure to different concentrations of Oxyphylla A for 24 h, the numbers of viable and damaged PC12/A53T-α-syn cells were detected by MTT and LDH reagents, respectively. As shown in the results (Fig. 1B), Oxyphylla A had no effect on cell viability at concentrations ranging from 10 to 300 µM. A similar result (Fig. 1C) with respect to cell death induced by Oxyphylla A was observed in the LDH-based cytotoxicity evaluation. We next assessed the potency of Oxyphylla A in terms of promoting the degradation of α-syn. PC12 cells stably transfected with WT, A30P or A53T SNCA genes were stimulated with Dox (1 µg/ml) for 24 h and treated with or without the indicated concentrations of Oxyphylla A for an additional 24 h. As revealed by the results (Fig. 1D-F), Dox significantly induced the expression of WT, A30P or A53T α-syn in PC12 cells compared to cells not treated with Dox. However, the levels of WT, A30P and A53T α-syn obviously decreased in a concentration-dependent manner when the cells were treated with Oxyphylla A. It is well-recognized that the A53T mutant form of α-syn plays an important role in neuronal dysfunction. Therefore, PC12/A53T-α-syn cells were used during further investigation. The level of A53T α-syn following treatment with Oxyphylla A (100 µM) at different time points was then assessed. The result (Fig. 1G) showed that Oxyphylla A decreased the level of A53T α-syn in Dox-inducible PC12 cells in a time-dependent manner. To demonstrate whether Oxyphylla A could degrade different forms of A53T α-syn, the Triton-soluble and Triton-insoluble forms of A53T α-syn in Dox-inducible PC12 cells were extracted and analyzed. Noticeably, both Triton-soluble and Triton-insoluble forms of A53T α-syn in Dox-inducible PC12 cells decreased in a dose-dependent manner after treatment with different concentrations of Oxyphylla A (Fig. 1H, I). Intriguingly, the level of the degradation of Triton-soluble A53T α-syn by Oxyphylla A was similar to that of Triton-insoluble A53T α-syn. Taken together, these findings suggest that Oxyphylla A is able to promote the degradation of WT, A30P and A53T α-syn in Dox-inducible PC12 cells without showing cytotoxicity.

**Figure 3.**
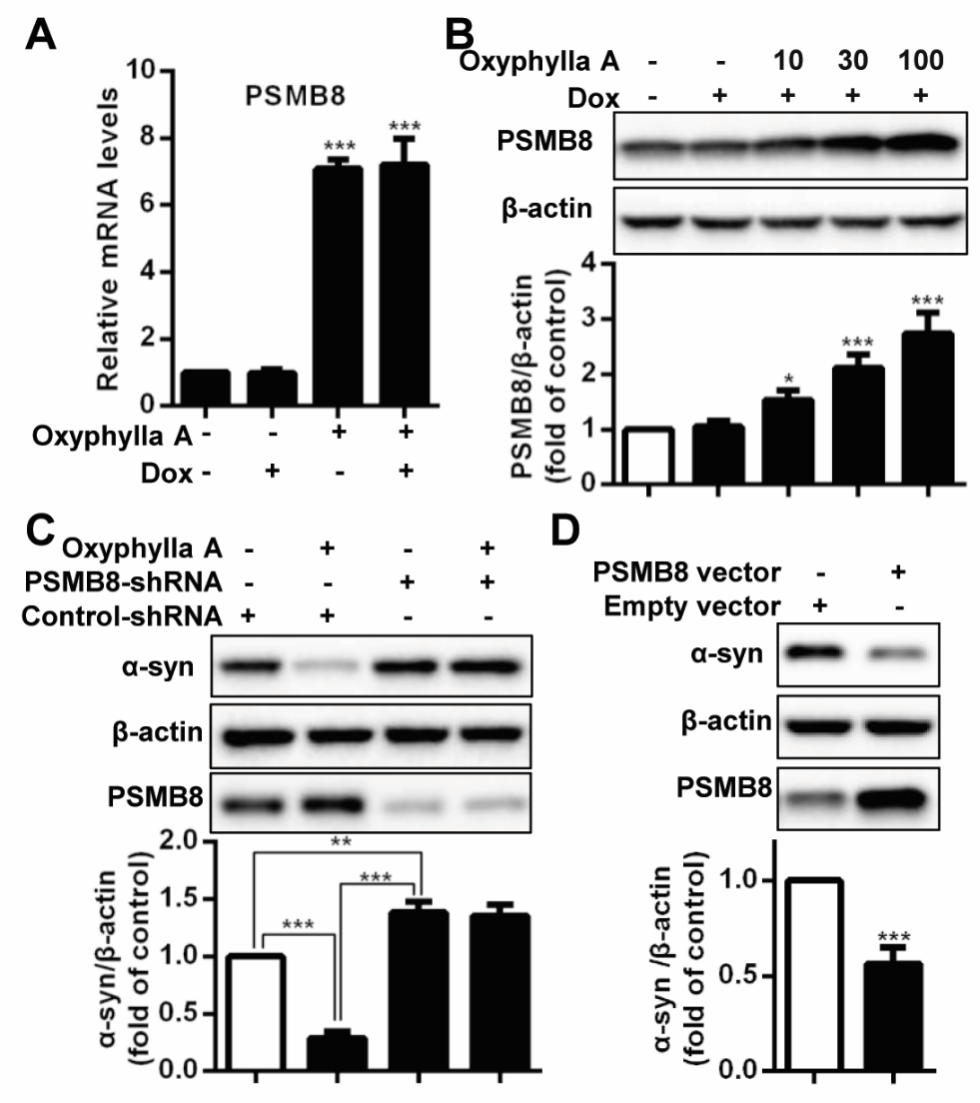
Oxyphylla A promotes α-syn degradation through upregulating the expression of genes encoding proteasome subunit beta type-8 (PSMB8). (A) PC12/A53T-α-syn cells were treated with 1 μg/ml Dox for 24 h and then with 100 μΜ Oxyphylla A for another 24 h. The levels of *PSMB8* mRNA were determined by qRT-PCR. (B) PC12/A53T-α-syn cells were treated with Oxyphylla A for 24 h and the protein level of PSMB8 was detected by Western blotting analysis. (C) PC12/A53T-α-syn cells were transduced by *PSMB8*-shRNA or control-shRNA, treated with Dox for 24 h and then treated with 100 μΜ Oxyphylla A for another 24 h. (D) PC12/A53T-α-syn cells were transfected with *PSMB8* or empty vector for 24 h and treated with 1 μg/ml Dox for another 24 h. Cell lysates were subjected to Western blotting analysis. Data are represented as mean ± SD of three independent experiments. *P < 0.05, **P < 0.01, ***P < 0.001, as compared to control group.

**Figure 4.**
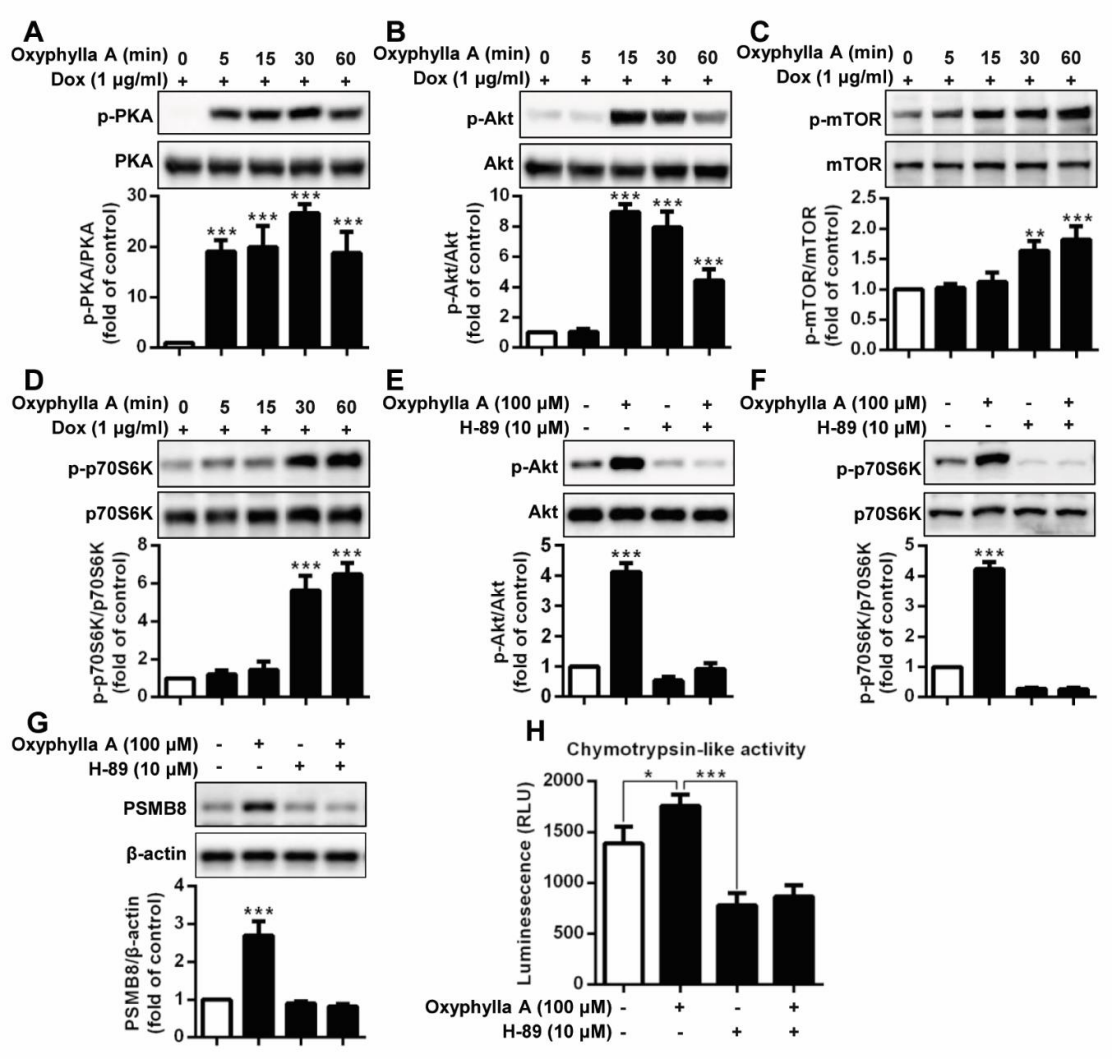
Oxyphylla A promotes *PSMB8* expression and enhances proteasomal activity through the PKA/Akt/mTOR pathway. Inducible PC12/A53T-α-syn cells were treated with 1 μg/ml Dox for 24 h and then with Oxyphylla A (100 μM) for the indicated time periods. The expression ratios of phosphorylated PKA/total PKA (A), phosphorylated Akt/total Akt (B), phosphorylated p-mTOR/total mTOR (C) and phosphorylated p-p70s6k/total p70s6k (D) were detected by Western blotting analysis. PC12/A53T-α-syn cells were pretreated with or without PKA inhibitor H-89 (10 μM) for 1 h and then treated with Oxyphylla A (100 μM) for 30 min. The expression ratios of phosphorylated Akt/total Akt (E), phosphorylated p70S6K/total p70S6K (F) were analyzed by Western blotting. PC12/A53T-α-syn cells were pretreated with or without PKA inhibitor H-89 (10 μM) for 1 h and then treated with Oxyphylla A (100 μM) for another 24 h. The protein levels of PSMB8 were determined by Western blotting (G). Cellular chymotrypsin-like activity was determined by specific bioluminogenic enzyme substrates (H). Representative Western blotting and data analysis of three independent experiments are shown. Data are represented as the mean ± SD. *P < 0.05, **P < 0.01, ***P < 0.001.

### Oxyphylla A selectively promotes α-syn clearance via the ubiquitin proteasome system

Previous studies have indicated that the degradation of α-syn was closely associated with the autophagy-lysosomal pathway and the UPS. Therefore, further investigation of the possible pathway involved in Oxyphylla A -induced α-syn degradation in inducible PC12/A53T-α-syn cells was carried out (Fig. 2A). After treatment with CQ, an autophagy inhibitor, at 30 µM, a visible increase in A53T α-syn levels in Dox-inducible PC12/A53T-α-syn cells was observed, suggesting that the inhibition of the autophagy-lysosomal pathway by CQ could suppress A53T α-syn degradation. However, a significant decrease in the level of A53T α-syn was observed after treatment with Oxyphylla A, indicating that the possible effect of Oxyphylla A was the promotion of A53T α-syn degradation. Accordingly, the UPS was then evaluated by the proteasome inhibitor MG132. Evidently, A53T α-syn degradation was markedly blocked in Dox-inducible PC12/A53T-α-syn cells treated with MG132 (0.1 µM), while no significant changes in A53T α-syn degradation were observed when the cells were subsequently incubated with Oxyphylla A. To further investigate the pathway by which Oxyphylla A promoted α-syn degradation, we detected autophagy markers (LC3 and p62). Western blotting analysis showed that Oxyphylla A had no effect on the levels of LC3 and p62 (Fig. 2B). These findings show that Oxyphylla A-induced A53T α-syn degradation is selectively mediated by the activation of the UPS. Protein degradation in the UPS has been considered to be facilitated by the cylindrical 20S proteasome core particle harboring caspase-, trypsin- and chymotrypsin-like activities. Consequently, luminescent assay was used to measure caspase-, trypsin- and chymotrypsin-like protease activities in Dox-inducible PC12/A53T-α-syn cells after Oxyphylla A treatment. The Dox-stimulated cells were treated with MG132, with or without Oxyphylla A (100 µM) treatment for 24 h. The caspase-, trypsin- and chymotrypsin-like protease activities were detected by three peptides Z-nLPnLD-aminoluciferin, Z-LRR-aminoluciferin and Suc-LLVY aminoluciferin, respectively. As shown by the results (Fig. 2C-E), after treatment with Oxyphylla A, chymotrypsin- and trypsin-like protease activities in Dox-inducible PC12/A53T-α-syn cells were significantly increased, while caspase-like protease activity showed no significant change. On the other hand, treatment with MG132 decreased trypsin- and chymotrypsin-like, but no caspase-like, protease activities. Moreover, the caspase-, trypsin- and chymotrypsin-like protease activities observed with MG132 treatment could not be reversed by treatment with Oxyphylla A, suggesting that Oxyphylla A-mediated protein degradation was attenuated by the UPS inhibition of MG132. Collectively, the findings revealed that Oxyphylla A selectively promotes A53T α-syn degradation by increasing chymotrypsin-like protease activity in the UPS without affecting the autophagy-lysosomal pathway.

### Oxyphylla A promotes α-syn degradation by upregulating the expression of the gene encoding proteasome subunit beta type-8 (PSMB8)

Considering the effect of Oxyphylla A on proteasome activities in the cylindrical 20S proteasome core particle, further evaluation of a panel of related genes was conducted using qRT-PCR. Among the tested genes, proteasome subunit beta type-8 (*PSMB8*) gene, known to code the product of the 20S proteasome subunit β5i, was remarkably amplified after treatment with Oxyphylla A in PC12/A53T-α-syn cells (Fig. 3A). This result was consistent with Oxyphylla A increasing chymotrypsin-like activity. Additionally, the resulting protein level of PSMB8 after treatment by Oxyphylla A was investigated. As shown in Fig. 3B, Oxyphylla A increased the level of PSMB8 proteins in a dose-dependent manner, leading to the upregulation of chymotrypsin-like β5i proteasome activity. To further illustrate the role of *PSMB8* in Oxyphylla A-induced A53T α-syn degradation, we employed a *PSMB8* shRNA knockdown assay and a *PSMB8* overexpression assay. In the *PSMB8* shRNA knockdown assay, PC12/A53T-α-syn cells transitionally transfected with *PSMB8* shRNA or control shRNA plasmids were treated with Oxyphylla A at 100 µM for 24 h (Fig. 3C). Notably, the degradation of A53T α-syn by Oxyphylla A was observed in cells transfected with control shRNA. Conversely, an accumulation of A53T α-syn in cells was triggered by silencing the *PSMB8* gene. However, the accumulation of A53T α-syn could not be reversed by treatment with Oxyphylla A. In addition, the overexpression of PSMB8 promoted the degradation of A53T α-syn (Fig. 3D). Overall, these findings demonstrate that Oxyphylla A exhibits a significant ability to increase PSM8 expression, leading to the upregulation of chymotrypsin-like proteasome activity, eventually resulting in the degradation of A53T α-syn.

### Oxyphylla A promotes PSMB8 expression and enhances proteasomal activity through the PKA/Akt/mTOR pathway

Recently, evidence has shown that protein kinase A (PKA) is a key regulator of proteasome activity [42], in that increasing the activity of PKA rapidly enhances proteasome assembly and activity levels. Therefore, we next determined the activity level of PKA after stimulation with Oxyphylla A. Figure 4A shows that Oxyphylla A significantly increased the phosphorylation of PKA in PC12/A53T-α-syn cells in a time-dependent manner. Moreover, the Akt/mTOR signaling pathway was also correlated with proteasome activity. An examination of the activity of Akt/mTOR signaling showed that Oxyphylla A time-dependently induced Atk phosphorylation and mTOR phosphorylation (Fig. 4B, C). mTOR stimulates p70S6K, which subsequently activates ribosomal S6k, which is involved in protein synthesis during mRNA translation. A similar time-dependent increase in p-p70S6K was observed when the cells were stimulated by Oxyphylla A (Fig. 4D). To investigate the potential correlations between PKA and Akt/mTOR/p70S6K, and the potency of the effect of Oxyphylla A on this pathway, a PKA inhibitor, H-89, was used. As shown in Fig. 4E, the phosphorylation of Akt was inhibited when the cells were pretreated with the PKA inhibitor H-89. Oxyphylla A was able to increase Akt phosphorylation in cells without PKA inhibitor H-89 pretreatment. However, the downregulation of Akt phosphorylation in the cells pretreated with the PKA inhibitor H-89 could not be reversed even with the addition of Oxyphylla A. Similar results were found regarding the regulation of 70S6K phosphorylation when the cells were pretreated with H-89 with the addition of Oxyphylla A (Fig. 4F). Levels of PSMB8 and chymotrypsin-like proteasome activity were consecutively analyzed by treatment with the PKA inhibitor H-89. The results were comparable to previous results, in that Oxyphylla A increased PSMB8 levels and chymotrypsin-like proteasome activity in cells without pretreatment with the PKA inhibitor H-89, rather than in cells pretreated with the PKA inhibitor H-89 (Fig. 4G, H). Together, these findings suggest that PKA might be the upstream regulator of Akt/mTOR/p70S6K signaling in ubiquitin proteasomal degradation, and Oxyphylla A might activate *PSMB8* gene expression and chymotrypsin-like proteasome activity *via* the regulation of PKA/Akt/mTOR/p70S6K signaling.

**Figure 5.**
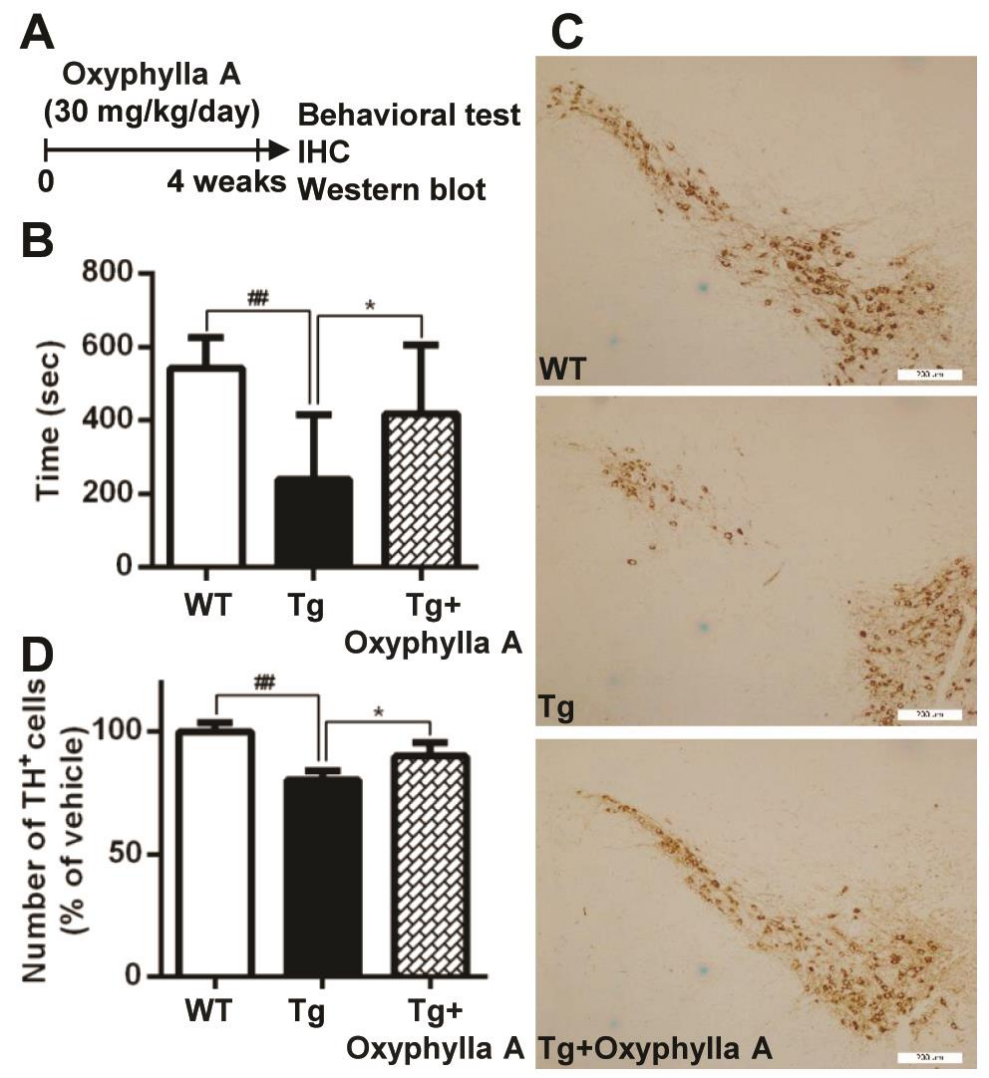
Oxyphylla A protects mice against α-syn-induced neurotoxicity. (A) Experimental scheme. Ten-month-old WT or homozygous A53T human α-syn Tg mice were gavaged daily with Oxyphylla A (30 mg/kg) or olive oil (0.2 ml) for 4 weeks. (B) In the rotarod test, the time spent on the rotating drum before falling was recorded and analyzed for each mouse (n = 10). (C) Representative images of tyrosine hydroxylase positive (TH^+^) neurons in the SNc (n = 5). (D) Quantification of TH^+^ cells in the SNc (n = 5). Data are represented as means ± SD. ^##^*P* < 0.01, compared to the WT group; *P < 0.05, compared to Tg group.

**Figure 6.**
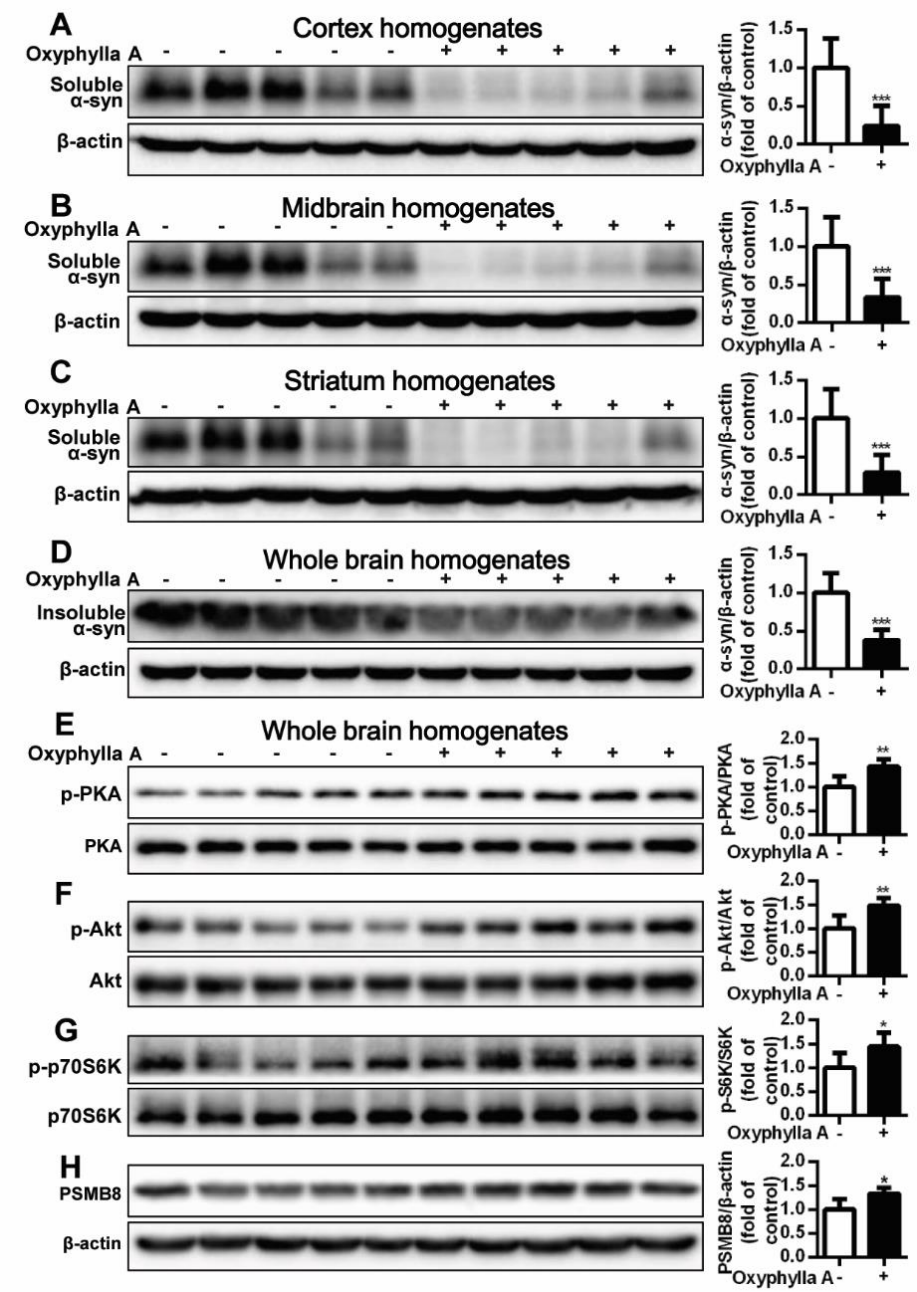
Oxyphylla A promotes the degradation of Triton-soluble and Triton-insoluble α-syn in A53T α-syn Tg mouse brains. Ten-month-old homozygous A53T human α-syn Tg mice were gavaged daily with Oxyphylla A (30 mg/kg) or olive oil (0.2 ml) for 4 weeks. The cortex (A), midbrain (B) and striatum (C) were homogenized and A53T α-syn was analyzed by Western blotting. (D) Whole brain levels of Triton-insoluble α-syn were analyzed by Western blotting and semi-quantified by densitometry. The expression ratios of phosphorylated PKA/total PKA (E), p-Akt/total Akt (F), p-p70S6K/total p70S6K (G) and the levels of PSMB8 (H) in whole brain were detected by Western blotting and semi-quantified by densitometry. Data are represented as means ± SD (n = 5). *P < 0.05, **P < 0.05, ***P < 0.001 compared to control.

### Oxyphylla A protects mice against α-synuclein-induced neurotoxicity

Next, an A53T α-syn transgenic (Tg) mouse model of PD was used to further evaluate the potential for the application of Oxyphylla A *in vivo*. Tg mice expressing mutant A53T human α-syn were given a daily treatment of Oxyphylla A (30 mg/kg) or the olive oil (0.2 ml) vehicle (10 mice per group) by gavage for 4 weeks (Fig. 5A). A rotarod test was employed to evaluate A53T α-syn Tg mouse behavior. The A53T α-syn Tg mice showed reduced locomotor activity compared to the WT mice in terms of the time spent on a rotating rod (Fig. 5B). In contrast, the reduced locomotor activity of Tg mice was noticeably overcome by treatment with Oxyphylla A. Moreover, the number of tyrosine hydroxylase (TH)-positive neurons was reduced in A53T α-syn Tg mice. In contrast, Oxyphylla A treatment conferred protection against the loss of dopaminergic neurons (Fig. 5C, D). Taken together, Oxyphylla A displays a protective effect against α-syn-induced neurotoxicity in A53T α-syn Tg mice.

### Oxyphylla A promotes the degradation of Triton-soluble and Triton-insoluble α-syn in Tg mice

In addition, Tg mouse brains were harvested, and A53T α-syn accumulation in different areas of the brain were detected. As shown in Fig. 6A-C, the accumulation of Triton-soluble A53T α-syn in the cortex, midbrain and striatum was dramatically reversed after treatment with Oxyphylla A for 4 weeks compared to that of the Tg mice treated with olive oil (control group). Likewise, the accumulation of Triton-insoluble A53T α-syn in the whole brain was markedly decreased by treatment with Oxyphylla A (Fig. 6D). Furthermore, the phosphorylated and total protein levels of PKA, Akt, and p70S6K and the levels of PSMB8 in the whole brain were analyzed by Western blotting. As shown in Fig. 6E, we found increased phosphorylation of PKA in brain tissues after Oxyphylla A treatment for 4 weeks compared with the control group. Similar variations in the phosphorylation of Akt (Fig. 6F) and p70S6K (Fig. 6G) were observed following Oxyphylla A treatment. Additionally, Oxyphylla A increased the level of PSMB8 in the whole brain compared with control mice.

**Figure 7.**
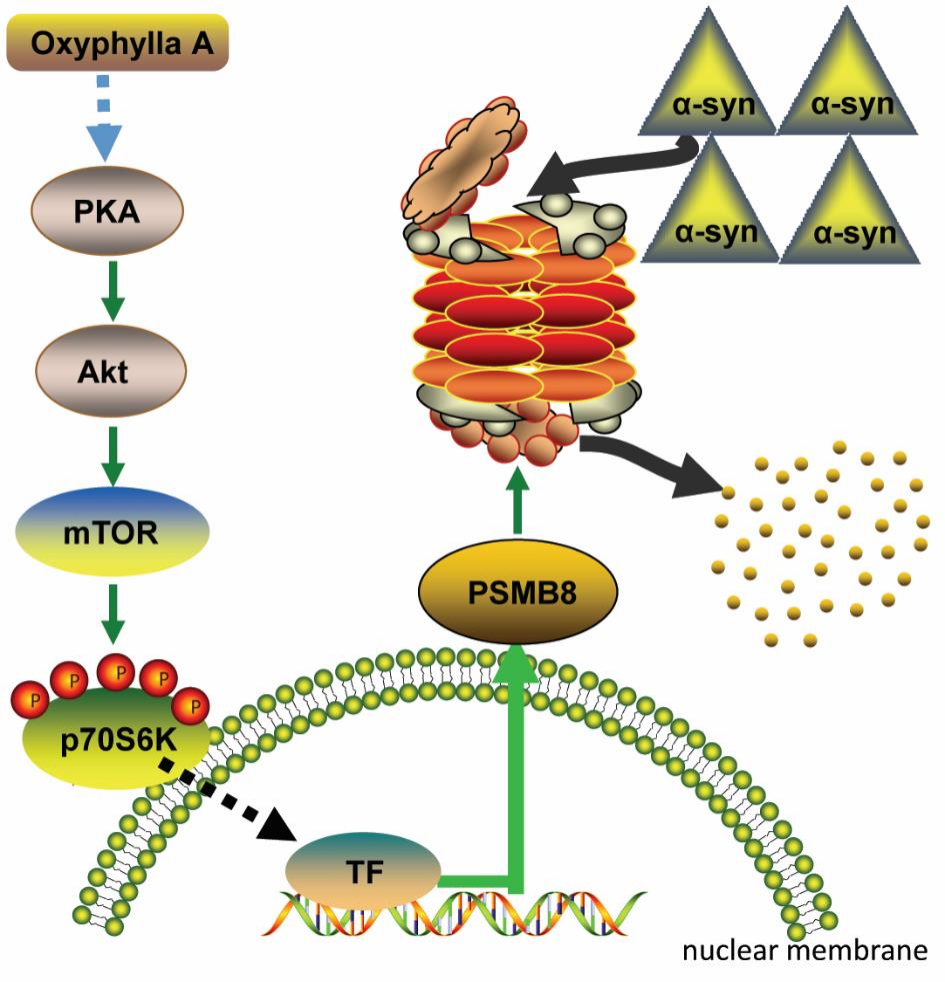
A proposed schematic of Oxyphylla A promotes degradation of α-syn *via* modulation of PKA/AKt/mTOR/PSMB8 pathway.

### DISCUSSION

In this study, Oxyphylla A was shown to significantly promote A53T α-syn degradation in Dox-inducible PC12/A53T-α-syn cells. The A53T α-syn degradation pathways, including the autophagy-lysosomal pathway and the UPS, were investigated. Oxyphylla A exhibited a selective mode of action, activating the UPS preferentially over the autophagy-lysosomal pathway. Moreover, chymotrypsin-like proteasome activity, rather than caspase-like or trypsin-like proteasome activities (involved in proteasomal degradation), was remarkably upregulated by Oxyphylla A treatment. Further investigation of gene expression indicated that Oxyphylla A preferentially increased *PSMB8* gene expression, which encodes the protein product of PSMB8, also known as chymotrypsin-like β5i. To further demonstrate the effects of Oxyphylla A on A53T α-syn degradation, the activities of PKA, Akt, mTOR1 and p70S6K were determined. The results showed that Oxyphylla A reduced A53T-α-syn accumulation by activating the PKA/Akt/mTOR/p70S6K signaling pathway *in vitro* and *in vivo*. Finally, an A53T α-syn Tg mouse PD model was employed to verify the potential utility of Oxyphylla A for PD treatment. Oxyphylla A markedly reversed the dysregulation of Triton-soluble and Triton-insoluble α-syn and displayed a promising protective effect against α-syn-induced neurotoxicity in A53T α-syn Tg mice.

Proteins in cells have important roles in cell structural maintenance, signaling transmission, small-molecule transportation and chemical reactions. However, in some cases, proteins such as α-syn will show misfolding and aggregation, causing pathological changes in cells, tissues or organs. Therefore, by maintaining protein homeostasis and minimizing the danger that misfolded and aggregated proteins pose to the cells, the UPS and the ALP constitute the major workhorses of the cell system. In this study, we found that Oxyphylla A selectively activated the UPS compared to the ALP under the influence of the proteasome inhibitor MG132 or the autophagy inhibitor CQ in an inducible PC12/A53T-α-syn cell model. Previous research on the potential role of the UPS in α-syn degradation showed that α-syn in neuronal cells accumulated upon proteasomal inhibition [18]. In our results, A53T-α-syn was observed to be degraded by Oxyphylla A treatment. We also examined the role of Oxyphylla A on the degradation of A30P and WT α-syn and found that Oxyphylla A promoted the degradation of both types in a dose-dependent manner (Fig. 1D-F). Moreover, A53T, A30P and WT α-syn have been reported to be degraded by the UPS system [19], and it is reasonable to conclude that Oxyphylla A promoted the different types of α-syn degradation by enhancing UPS activity. The UPS system is known to be responsible for the degradation of monomeric or small oligomeric species of α-syn (together with the CMA), while macroautophagy is usually linked to large aggregates. Considering that the dynamic balance between mono/oligomer/aggregates may be disrupted when mono and oligomer α-syn types are degraded, there would not be enough material to build up the aggregate species. Aggregated α-syn levels would decrease accordingly. A study showed that the enhancement of proteasome activity by PKA activation can reduce protein aggregation which is normally Triton X-100 insoluble [43]. Therefore, a significant reduction in Triton-soluble and Triton-insoluble fractions when using Oxyphylla A (Fig. 1H, I) can be observed. Proteasomes are large complexes with various proteolytic activities that contain three major complexes: the 19S regulatory particle, the 20S core particle forming the core of the 26S proteasome and the 11S regulatory particle. The 20/26S proteasomal machinery is among the most important components with respect to the clearance of intracellular proteins [44]. Previous data have indicated that α-syn mutants showed a faster rate of fibrillation (A53T) and amorphous aggregation of soluble oligomers (A30P) than WT-α-syn [8]. However, the mechanism for the UPS degradation of A30P and A53T is the same as that reported [19]. Our data showed that Oxyphylla A promoted the dose-dependent degradation of A30P and A53T α-syn which was very similar to WT. In additoin, Oxyphylla A increased the proteolytic activity of the 20S proteasome, particularly chymotrypsin-like proteasome activity, leading to A53T-α-syn clearance. Moreover, increasing gene and protein expression levels of PSMB8, also known as immunoproteasome subunit β5i (which has "chymotrypsin-like" activity), after Oxyphylla A treatment was detected by qRT-PCR and Western blotting. However, previous studies reported that stable expression of both wild-type and mutant α-syn in PC12 cells resulted in the prominent impairment of chymotrypsin-like 20S/26S proteasomal protein cleavage [44]and proteasome inhibition in human α-syn Tg mice as they aged [45]. In this study, we used an inducible cell line transiently expressing α-syn and the short-term overexpression of α-syn might not dramatically affect chymotrypsin-like activity, as a reduction trend was detected. In addition, with the extended Dox induction time, a dramatic inhibition of chymotrypsin-like activity after 1 week was observed (data not shown), which was consistent with previous reports. To elucidate the potential signaling pathway regulating A53T-α-syn clearance in the UPS modulated by Oxyphylla A, certain proteins, such as PKA, Akt and mTOR were examined. Recent investigations showed that the activation of PKA enhanced the phosphorylation of Rpt6, a component of the 19S proteasome complex, which, in turn, led to enhanced proteasome activity and the suppressed aggregation of proteins [46, 47]. However, Oxyphylla A elevated PKA phosphorylation activity without affecting the phosphorylation of Rpt6 (data not shown). Serial studies revealed a connection between Akt signaling and protein degradation [48]. Moreover, recent findings suggest that the activation of the mechanistic target of Rap complex 1 (mTOR) promotes not only protein synthesis, but also an increased capacity for protein degradation [49, 50]. Our Western blotting and shRNA knockdown assays also showed that Akt, mTOR and the effector of the mTOR kinase complex, p70S6K, were activated by Oxyphylla A, with an increase in the expression of the *PSMB8* gene encoding protein containing chymotrypsin-like proteasome activity. Our results provided further confirmation that the activation of mTOR elevated the levels of intact and active proteasomes through an increase in the expression of genes encoding proteasome subunits. As a consequence, our findings provide a logical basis for the hypothesis that Oxyphylla A promotes the degradation of A53T-α-syn by enhancing the PKA/Akt/mTOR/PSMB8 pathway. However, the signaling of the activated Akt/mTOR/p70S6K pathway induced by Oxyphylla A transmitted from the cytoplasm to the nucleus remains puzzling. Previously, we reported that Oxyphylla A exerts neuroprotective effects against MPTP-induced PD *in vitro* and *in vivo *[34]. Herein, we illustrated a further potential application of Oxyphylla A in the degradation of A53T-α-syn for the treatment of PD. Our WT and homozygous A53T human α-syn Tg mouse model results revealed that Oxyphylla A displayed a promising ability to reverse the accumulation of A53T-α-syn in the mouse brain and to rescue the pathologically induced reduction in locomotor activity on a rotating rod and the decrease in TH-positive neurons.

Herein, we proposed a possible mechanism (Fig. 7) of the neuroprotective effect of Oxyphylla A in a PC12/A53T-α-syn cell model and in A53T α-syn Tg mice. Oxyphylla A activated the phosphorylation of PKA, subsequently activating Akt and mTOR. The activation of mTOR induced a signaling transition from the cytosol to the nucleus, leading to selective triggering of *PSMB8* gene expression. The increased expression of PSMB8 in the UPS specifically impelled chymotrypsin-like proteasome activity, resulting in promotion of A53T α-syn degradation. This is the first report on the neuroprotective effect of Oxyphylla A as a possible treatment for PD *via* modulation of the PKA/AKt/mTOR/PSMB8 pathway.

In the past few years, many therapeutic approaches, such as the use of small-molecule compounds, gene therapy and treatment with fetal or retinal dopamine-synthesizing cells or growth factor synthesis-supporting formulations, have failed in clinical trials due to undesirable side effects [51]. Recently, many studies have focused on the neuroprotective ability of natural products for PD therapy [33]. Accumulating evidence indicates that natural products from TCM may constitute an alternative therapy for PD by targeting the ALP. Oxindole alkaloids from *Uncaria rhynchophylla* (Miq.) Jacks (Gouteng), conophylline, polyphenols, smurensin G, triterpene, flavonoids, disaccharides and resveratrol were shown to attenuate cellular toxicity in a PD model by inducting autophagy [33]. Here, for the first time, we showed that a natural product, Oxyphylla A, protected against neurotoxicity both in a PC12/A53T-α-syn cell model and in an A53T α-syn Tg mouse model by promoting proteasome activity.

### Conclusions

Taken together, the results of the present study underline the unexplored potential of Oxyphylla A as a potential anti-PD candidate. Oxyphylla A exhibits a protective effect against A53T-α-syn-induced neurotoxicity both *in vitro* and *in vivo*. Further exploration of the molecular mechanism underlying the activation of the PKA/Akt/ mTOR/PSMB8 signaling pathway by Oxyphylla A, combined with rational structural modifications, could enhance the potency of Oxyphylla A in the micromolar to nanomolar range. Critically, this is the first demonstration that Oxyphylla A promotes A53T-α-syn degradation by activating the PKA/Akt/ mTOR/PSMB8 signaling pathway, resulting in the rescue of the neurotoxicity induced by accumulated A53T-α-syn *in vitro* and *in vivo*, with potential therapeutic application in PD treatment.

## Supplementary Materials

The Supplemenantry data can be found online at: www.aginganddisease.org/EN/10.14336/AD.2019.0612.
