# Supplementary material for: Oxyphylla A Promotes Degradation of α-Synuclein for Neuroprotection via Activation of Immunoproteasome
Source: Aging Dis. 2019 Jun 12;11(3):559–74. doi: 10.14336/AD.2019.0612 (PMC7220298; doi:10.14336/AD.2019.0612)
Supplement: Supplementary file 1 — The Supplemenantry data can be found online at: www.aginganddisease.org/EN/10.14336/AD.2019.0612. [file AD-11-3-559-s.pdf]

SUPPLEMENTARY DATA

Oxyphylla A Promotes Degradation of α-Synuclein for Neuroprotection *via* Activation of Immunoproteasome

Hefeng Zhou[1,#], Shengnan Li[1,#], Chuwen Li[1], Xuanjun Yang[1,2], Haitao Li[1], Hanbing Zhong[2], Jia-Hong Lu[1,*], Simon Ming-Yuen Lee[1,*]

[1]State Key Laboratory of Quality Research in Chinese Medicine and Institute of Chinese Medical Sciences, University of Macau, Macao, China.
[2]Department of Biology, South University of Science and Technology, Shenzhen, China.

© 2019. Zhou H et al. Published online at http://www.aginganddisease.org/EN/10.14336/AD.2019.0612

SUPPLEMENTARY DATA

Supplementary Table 1. PCR primers.

Name	Forward primer	Reverse primer (5'→3')
PSMA1	CGCTGAAGAGAGCACAGTCA	CATCAGCAGTTAGACCCGCA
PSMA2	CGGCATGGGTCCAGATTACA	ATGGACGAACACCACCTGAC
PSMA3	GCTCCATTGGCACTGGGTAT	ACCCCAAACACAACACCATCT
PSMA4	CAGTTTGGAGGCAAACGTCC	TCCATCCCCCGTAGTTTCCA
PSMA5	ATCTGGCTTTGCAGTTCGGA	CCATGTGAAACAGTTGGGGC
PSMA6	AGGGCCGACTCTACCAAGT	GGAATCCAGTAGTTTGTCAG
PSMA7	TCTGAAGCAGCGTTACACAC	ATGGTATGTGCCTGAGGGGT
PSMA8	GCAAATGCAATAGGCCGGAG	CCAGACTGCACAACTTCAAG
PSMB1	ATGCCTTCAACGGAGGTACT	CCGAGTGTCTGAAGCAACGA
PSMB2	ATGAATTGTCCCCCACAGCA	GTTCACATGATAAGGGGTC
PSMB3	GCTTGGACCCGAAGACCTTT	TGCTCTGGATCCATGTTGGG
PSMB4	GCCAGATGGTGATTGATGAG	AGAGGGTTCATCTTGGAGCG
PSMB5	TGGCCTTCAAGTTTCAGCATG	TGTCTGGGAAGCAATGTAGG
PSMB6	TTCACTGCCAATGCTCTTGC	ATCCTAGGCTTCAGGGAGG
PSMB7	CGAAAGCTCGGAAAACTGGC	CCCAGCACCACAGCAATAAAT
PSMB8	TGGCGTTACTGGATCTGTGC	CCTTAGGAATTCAGTGGGCT
PSMB9	CGCATCTACTGTGCCCTCTC	TCCTCCAGTTCCAACCCGTG
PSMB10	CGCCCCCAAAATCTACTGCT	CTGACGCAAGATACGGGTGA
PSMC1	TTATCAGACCAGGCCGCATT	ACATCATCAGCCAGCGTCAT
PSMC2	TCCTCGGGGCAACATCAAAG	TGCGTTCGACCCTCTAAGTC
PSMC3	TGCTGGATGTTGACCCCAAT	CAGGAAGTACGTTTGTCGGGT
PSMC4	CGTCGCCAGAAGAGGTTGAT	ACAGCCAACATTCCACTCTCC
PSMC5	AAAAGTTCGTCTGTTGCGGG	AGGATGGACCTTGACCAACAC
PSMC6	GCTTCAGGACTACCGCAAGA	GCACTTCGCCTACAATCTGTC
PSMD1	AAACACCAGACGCTAGTCCA	TGCAGGTGTAACTCGATAGC
PSMD2	TCGGAATGAGTGCGATCCTG	AGGCCAAACCTAGTCCGAAG

© 2019. Zhou H et al. Published online at http://www.aginganddisease.org/EN/10.14336/AD.2019.0612

SUPPLEMENTARY DATA

PSMD4	TTGGCCCTGGCCCTTCG	TCGTTCACCTTCAGTCCCAG
PSMD5	CTTTGAACGCCTCCTCACGA	CTGCTGCTCAGGTGATAGGT
PSMD6	CCAGTCGTTAGCGGTCGTAG	CCACACCAAATGCTTCTGCC
PSMD7	TGGAGCACTTGTTACGGGAC	TGTGGTGGTTGATGGGTAGC
PSMD8	GGACAACCGGCGTTTCAAG	GCTCCAGCAGAACCAACTTC
PSMD9	CAGCCAGTATTGCGGGCCT	CTCTGCGGATCACCATGACA
PSMD10	GGAGGGGTGTGTGTCTAACC	AATGCTGTTCTGCTGTCCTGAT
PSMD11	TGAAAAGGCCCTGACAGACT	GGGAGATTCTGACCTGTACTC
PSMD12	GATTCCCAAATACAAGGATC	CCGTAGCTCCACCCCATAGT
PSMD13	GCAGGCTACAAAGGAAACCA	CTACACAGCCCAGAAACCGT
PSMD14	ATGCCACAATCAGGAACTGG	ACAACCATCTCGGGCCTTC
PSME1	TTCGAGCTGTGCTTTCGCT	CACGAAACACATCCACCTTG
PSME2	AGCCCGTAAACAGGTGGATG	GGTCAGCCACATTGAGGGAA
PSME3	AAGAAGCGCAGGTTGGATGA	GAACCCACATTTTGACCGTGT
PSME4	CAGAGAAAGGATAGGGAGTG	ATGTTGTTGGAGCAGTATTTG
PSMF1	ATTCCCTAATCAGGCATGGGC	TGTTTGCGGCTTCTACATCAG
GAPDH	GACATGCCGCCTGGAGAAAC	AGCCCAGGATGCCCTTTAGT

© 2019. Zhou H et al. Published online at http://www.aginganddisease.org/EN/10.14336/AD.2019.0612
